# Validation of the Parent Health Locus of Control Scales in an Italian sample

**DOI:** 10.1186/1824-7288-35-13

**Published:** 2009-05-19

**Authors:** Sabrina Bonichini, Giovanna Axia, Marc H Bornstein

**Affiliations:** 1 Department of Developmental Psychology and Socialization, University of Padua, 35131, Padova, Italy; 2 Eunice Kennedy Shriver National Institute of Child Health and Human Development, NIH, Bethesda, Maryland 20814, USA

## Abstract

**Aim:**

This study examined the psychometric properties and the replicability of De Vellis's (1993) Parent Health Locus of Control (PHLOC) scales in an Italian sample.

**Method:**

The Italian version of PHLOC was administered to 470 mothers of birth to 3-year-old children.

**Results:**

Varimax rotated confirmatory factor analysis identified the six original subscales, namely Child, Divine, Fate, Media, Parental, and Professional influences over child health. Internal consistency of the subscales was good (α > .70), and test-retest correlations were adequate (*r *> .80). More highly educated mothers rated media influence as most important. No differences were found considering children's age, gender, and birth order.

**Conclusion:**

The results offer good evidence of the cross-cultural validity of the PHLOC scales, an instrument that can be useful in interventions with families by the health care practitioners to prevent neglectful childrearing practices.

## Background

Health locus of control is defined as the set of beliefs a person has about his or her personal influence on health. This set of beliefs includes: internal locus of control (if the individual believes that personal actions or thoughts can affect their outcomes) and external locus of control (if the outcome is believed to be determined by powerful others, such as God, health professionals, or if chance is believed to control the outcome) [1,2]. Empirical research suggests that health locus of control plays a significant role in determining people's health-related behaviours [3].

Individuals' health locus of control influences their own health-related behaviours, including health risk behaviour and adherence to recommended health care regimens [4,5]. For example, adolescents with high internal control are more likely to abstain from risky behaviors such as smoking and drinking [6], and children suffering for asthma who perceive their ability to control their health more positively adhere to their recommended regimen [7]. Perrin and Shapiro [8] studied differences among children with and without a chronic physical illness, and especially among their parents; they found that learning occurs from experiences related to illness, resulting in an increased external orientation in locus of control beliefs. They concluded that health care professionals should be made aware of the tendency for young children, and for children with a chronic illness and their families, to rely heavily on providers, perhaps to the detriment of learning effective skills for independent health-related decision making.

Parents have an important role in the promotion of their children's health especially when their children are very young. It is therefore of interest to assess parental locus of control relative to children's health [9]. Parents are typically the primary agents in promoting their children's health, giving direct care, providing access to health services, modeling attitudes and behaviors that influence their children's well-being, and instilling a lifelong orientation in health behaviors and future morbidity [2,10,11]. In consequence, we studied locus of health control in parents of young children.

Taken together, this literature suggests that children's compliance with interventions may need to include components aimed at enhancing health locus of control both in parents and children themselves and that health locus of control can be considered a factor that promotes adherence to recommendations related to preventive medicine. Given the importance of locus of control in health-related situations, we asked how it should best be assessed.

The PHLOC is a major instrument used to evaluate adult and parent health locus of control. The goal of the present study is to verify the replicability and psychometric properties of the factor structure of the Parent Health Locus of Control (PHLOC) [2] in an Italian sample of mothers of newborn to 3-year-old children. Because of the significance of the PHLOC, validation studies in other cultures are requisite to verify the robustness of the instrument. More generally, assessment instruments in health research must be validated by investigators using different samples in different contexts.

## Method

### Participants

470 mothers of healthy, normally developing children, aged birth to 39 months (M = 16.64; *SD *= 8.98), 49.8% females (*n *= 234) and 57.9% firstborn (*n *= 272), were recruited in 6 different cities of northern-central Italy with the help of pediatricians of the National Health Services and teachers at nursery schools. The mothers were all married, 72.4% (*N *= 340) employed, and they had a mean of 13.5 years of education (*SD *= 3.33, *range *8–18).

### Procedure

Mothers were contacted directly by the researchers who collected the data and who explained briefly the goals of the study during an appointment at their homes.

### Measures

The Parent Health Locus of Control [2] is a 30-item questionnaire used to assess a parent's beliefs about the health of a child. The questionnaire assesses beliefs of Child (6 items), Divine (4 items), Fate (5 items), Media (4 items), Parental (6 items), and Professional influences (5 items) over child health. In particular, the Child subscale assesses the extent to which parents think their child directly controls her/his health (e.g., *My child is in control of her/his health*); the Divine subscale assesses parental beliefs about the importance of God in influencing child health (e.g., *My child's wellbeing is in God's hands*); the Fate subscale provides an index of the extent to which parents believe that the health status of their child is predominantly a matter of luck (e.g., *Whether my child avoids injury is mostly a matter of luck*); the Media subscale is a measure of how much parents believe that all media, such as TV, magazines, and books, can directly influence their child's well-being (e.g., *What my child sees on TV programmes can affect her/his health)*; the Parental subscale evaluates how much parents feel they are the principally responsible for their children's health (e.g., *I have the ability to influence my child's well-being)*; the Professionals subscale estimates the extent to which parents think that health professionals control their children's health (e.g., *Health professionals keep my child from getting sick)*. Parents were asked to express their degree of agreement or disagreement with each statement using a 6-point Likert scale with 1 = *strongly disagree *and 6 = *strongly agree*. Administration requires about 10 min. For each dimension a mean score is calculated by summing all the values for each statement/item belonging to the subscales and so ranges from 1 to 6 for every subscale. The American standardization [2] showed internal consistency reliability coefficients (α) above .70 for all scales and test-retest (*r*) correlations all above .60. The original version of the PHLOC was forward-translated into Italian by a bilingual researcher and then back-translated by another bilingual researcher. The two versions were compared according to back-translation techniques [12]. In the Italian version, two of the 30 items were eliminated as not appropriate for mothers of children under 3 years of age (*My child can decide to live a safe and healthy life*, and *My child reads influence his/her well-being*). A pilot study with a small number of mothers ensured that the instrument was comprehensible for non-professionals.

## Results

Means and standard deviations for each subscale are reported in Table 1. The results from a principal component factor analysis using Varimax rotation showed that seven factors could be extracted according to Kaiser's rule. However, the seventh factor was saturated only by three items and its eigenvalue was 1.1, only marginally above the criterion value of 1, explaining a marginal proportion of variance (4.1%). The Scree plot showed six factors. Items were grouped according to the sub-scale structure proposed by De Vellis et al. [2]. Only one item loaded on two factors simultaneously: *My child's safety depends most on what my child does*. loaded both on Fate (.25) and on the original Child factor (.30). The six factors that emerged from factor analysis explained 59.21% of total variance (Table 1).

**Table 1:** Varimax rotated factorial analysis of 28 PHLOC items (*N *= 470): item factor loadings, communality, Eigenvalues, percentages of explained variance, Cronbach's alphas, scale means and standard deviations for each factors

Item no.	CHILD	DIVINE	FATE	MEDIA	PARENTAL	PROFESSIONAL	Item communality
4	.66						.48
9	.73						.60
15	.30				.25		.36
25	.82						.72
28	.78						.64
11		.88					.82
16		.91					.87
23		.87					.81
7			.66				.51
12			.79				.65
19			.81				.73
27			.76				.72
30			.63				.64
6				.82			.72
8				.81			.71
14				.70			.60
2					.16		.60
13					.71		.54
18					.75		.62
20					.67		.53
24					.66		.48
26					.85		.74
29					.74		.56
1						.31	.67
3						.53	.67
5						.72	.58
10						.68	.51
21						.69	.57
Eigenvalue	1.96	2.36	3.44	1.4	5.77	1.61	
% expl.	6.99	8.44	12.3	5.00	20.63	5.75	
α	.76	.91	.82	.74	.80	.72	
Scale *M*	2.87	3.07	2.64	3.32	4.9	3.55	
Scale *SD*	.92	1.57	1.05	1.19	.70	.94	

The internal consistency of the subscales was good, αs = .72 to .91. Correlations among the PHLOC scales (Table 2) showed that the scales are moderately independent of each other.

**Table 2:** Pearson correlations coefficients among the PHLOC scales (*N *= 470)

	DIVINE	FATE	MEDIA	PARENTAL	PROFESSIONAL
CHILD	.22**	.36**	.34**	.25**	.32**
DIVINE		.42**	.11*	.11*	.21**
FATE			.09*	.04	.29**
MEDIA				.25**	.11*
PARENTAL					.25**

* *p *< 0.05 (2-tails); ** *p *< 0.01 (2-tails)

Test-retest reliability was assessed in a subgroup (*n *= 35) after 2 months. Pearson correlation reliability coefficients were: .87 for Child, .98 for Divine, .87 for Fate, .84 for Media, .98 for Parental, and .96 for Professional scales.

To evaluate possible links between sociodemographic characteristics of the sample and scores for each PHLOC subscale, Pearson correlations were calculated. Table 3 shows small but significant negative correlations between the level of mothers' education and occupation and their responses to the Divine, Fate, and Professional subscales. The higher the parent level of education and occupation, the lower the influence of God, destiny, and health professionals in health related events of their children. The correlation between mothers' years of education and mass media influence was positive: the more education, the higher the influence of media on their child health-related beliefs.

**Table 3:** Pearson correlations coefficients between the PHLOC scales and sociodemographic variables (*N *= 470)

	Mother education	Mother occupation	No. of children
CHILD	.03	-.07	.02
DIVINE	-.15**	-.14**	.05
FATE	-.22**	-.07	.09
MEDIA	.10*	.07	-.11*
PARENT	,05	,04	,01
PROFFESS.	-.15**	-.15**	-.02

* *p *< 0.05 (2-tails); ** *p *< 0.01 (2-tails)

Finally, an Age (0–12 months, 13–24 months, 25–39 months) × Gender (boys vs. girls) × Birth order (firstborn vs. laterborn) analysis of variance was carried out for each PHLOC subscale. Analyses of variance yielded no significant effects for any subscale.

## Discussion

This work aimed to verify the psychometric properties and reliability of the factor structure of the Parent Health Locus of Control Scale [2] in an Italian sample of mothers of newborn to 3-year-old children. The results confirmed the original factor structure with the 28 items of the original instrument distributed among six theoretical dimensions as conceptualized by the original authors of the PHLOC (Child, Divine, Fate, Media, Parental, and Professional influences). Confirmatory factor analysis closely approached the standards for adequate fit for models of this type [13]. The internal consistency coefficients of the six scales were good, supporting the multidimensionality of the construct measured and the existence of different factors which parents perceive to influence their children's health.

The factorial similarity to the original U. S. American version of the PHLOC suggests good cross-national correspondence of the scales and supports the validity of the instrument. This consideration is reinforced by a previous study [14] that demonstrated similar results in a Norwegian sample of mothers of 2-year-olds. The present Italian study contributes to the robustness of this instrument across languages and cultures.

We did not find differences in mothers' attitudes about their children's health in relation to their children's age. Other studies have demonstrated that parental attitudes about child influences change with child age [15] in parents whose children attended elementary school. Age differences between the two studies might explain why we did not find child age effects.

Italian mothers attributed the most importance to themselves and to professionals in relation to their children's health. This is an encouraging result because parents' experience of control over their children's health is positively associated with their adopting safety measures [16].

In pediatric patients, the responsibility of parents for health-related behaviors of their children and the caregivers' impact on the development of children's own subjective health concepts have to be considered. Parents monitor their children's health state, decide whether medical care is to be sought, and comply with medical recommendations or not. Knowledge of family health beliefs may help to develop health promotion programmes, and investigating health locus of control can be useful in relation to the promotion of specific health behaviors and to individualize patient treatment based also on locus of control to enhance resilience [1].

Future research on how information derived from the PHLOC predicts parental behaviors regarding health-risk prevention in infants and toddlers is desirable. Finally, the PHLOC might prove useful in health education interventions with families to prevent neglectful childrearing practices.

## Competing interests

The authors declare that they have no competing interests.

## Authors' contributions

SB carried out the study, performed the statistical analysis and drafted the manuscript; GA and MHB participated in the design of the study and its coordination. All authors read and approved the final manuscript.
